# Routine use of indirect calorimetry in critically ill patients: pros and cons

**DOI:** 10.1186/s13054-022-04000-5

**Published:** 2022-05-05

**Authors:** Elisabeth De Waele, Arthur R. H. van Zanten

**Affiliations:** 1 Department of Clinical Nutrition, Vrije Universiteit Brussel, Laarbeeklaan 101, 1090 Brussels, Belgium; 2 Intensive Care Unit, UZ Brussel, Vrije Universiteit Brussel, Laarbeeklaan 101, 1090 Brussels, Belgium; 3 Department of Intensive Care Medicine and Research, Gelderse Vallei Hospital, Willy Brandtlaan 10, 6716 RP Ede, The Netherlands; 4 Division of Human Nutrition and Health, Chair Group Nutritional Biology, Wageningen University and Research, HELIX (Building 124), Stippeneng 4, 6708 WE Wageningen, The Netherlands

## Purpose of review

**Fig. 1:**
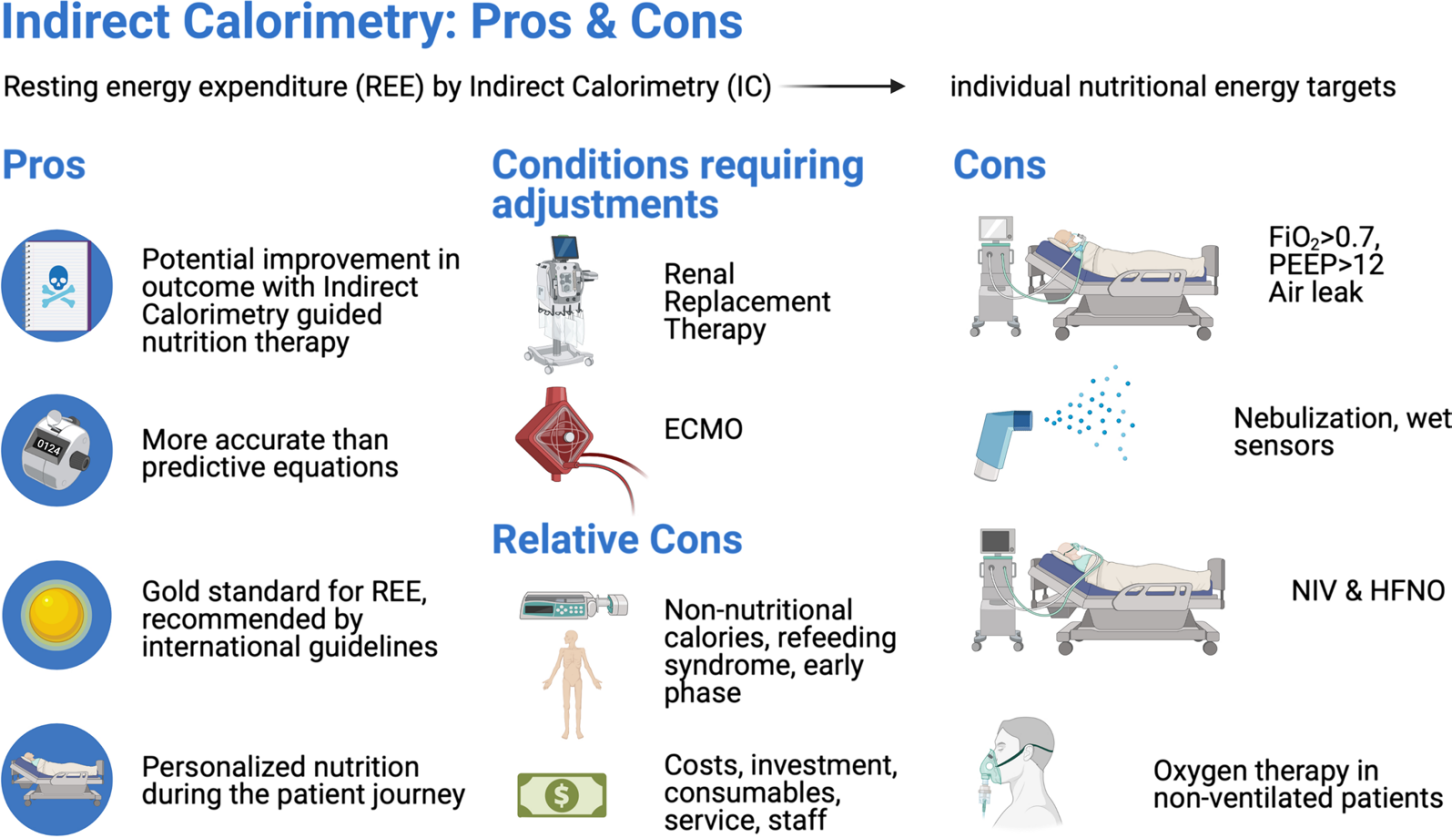
Indirect calorimetry: pros, (relative) cons and when adjustments are needed. Overview of benefits of indirect calorimetry technology, technical and clinical limitations, and situations when the measured resting energy expenditure should not be the target for total energy administration and adjustments are necessary. REE: resting energy expenditure, IC: indirect calorimetry, ECMO: extracorporeal membrane oxygenation, FiO_2_: fraction of inspired oxygen, PEEP: positive end-expiratory pressure, NIV: non-invasive ventilation, HFNO: high-flow nasal oxygen. Figure created with BioRender.com (license FS23TTD3HV)

To review the pros and cons of indirect calorimetry (IC) to estimate resting energy expenditure (REE) and define individual nutritional energy targets among critically ill patients. We evaluate pros, (relative) cons and when adjustments of REE are needed (Fig. 1).

## Introduction

IC measuring O_2_ consumption (VO_2_) and CO_2_ production (VCO_2_) represents real-time energy metabolism [1]. REE assessment by IC to guide nutrition therapy is recommended by international nutrition guidelines for adult ICU patients, although the level of evidence is still low (grade B recommendation) [2].

## Indirect calorimetry does not change a patient's outcome—Yes, it does

Indirect calorimetry is not lifesaving but a monitoring tool providing factual information on metabolism. Retrospective data revealed a U-shaped association between 60-day mortality and calorie intake in early critical illness, underlining the importance of targeting individual energy demands and preventing both overfeeding and underfeeding [3]. In a systematic review and meta-analysis in 2020, no improved outcomes were found when IC was used, although prescribed energy targets were more closely met when IC informed energy delivery compared with predictive equations [4]. The more precise intake observed suggests that IC feedback improves feeding performance, compared with equations, and may prevent underfeeding and overfeeding in individual patients.

In 2021, another meta-analysis showed a 23% reduction in short-term mortality when energy targets were based on IC [5]. In another meta-analysis, lower 28-day mortality was found when isocaloric IC-guided nutrition was compared with (hypocaloric) feeding protocols using predictive equations, however, without effect on nosocomial infections and 90-day mortality [6]. All meta-analyses did not find a shorter length of stay (LOS) or mechanical ventilation duration, possibly due to higher calorie intake leading to higher CO_2_ production, potentially prolonging mechanical ventilation duration and LOS.

## You can reliably estimate energy expenditure with predictive equations—no, you can't

Personalised nutrition should be based on individual energy targets. However, predictive equations are highly inaccurate, potentially leading to 500–1000 kcal/day nutrition targets higher or lower than individual demands, conferring marked risks of underfeeding and overfeeding [7]. Using only the VCO_2_ from mechanical ventilators to estimate REE is inferior to IC and barely better than predictive equations [8]. Responding to varying needs during the patient journey is recommended. IC using a canopy can be expanded to the post-ICU phase [9]. The ICALIC research group helped to develop moderate-cost highly efficient technology to measure REE in ventilated and spontaneous breathing patients [10].

## You can use it in all patients—no, you can't at any time

In ventilated patients with FiO_2_ > 0.7 and PEEP > 12 cmH_2_O, no reliable measurements of VO_2_ and VCO_2_ are possible. Furthermore, any air leak excludes the use of IC as not all exhaled gas will meet the device's sensors. Typical examples are pneumothorax, subcutaneous emphysema, or tracheal–oesophageal fistula. High-flow nasal oxygen therapy or non-invasive ventilation precludes IC use. As moisture affects the performance of sensors, nebulisation during measurement is not recommended. In non-ventilated patients, measurements can only be taken when oxygen therapy has been stopped [1]. As these limitations are present in many patients, equations are still needed then.

## You should give nutritional energy to meet the REE in all patients—no, you shouldn't

REE can be used to set energy targets until a new measurement has been taken. However, endogenous energy production can be marked in the early phase of critical illness (> 1000 kcal/day). Adding full nutrition to this non-inhibitable energy production may induce overfeeding [1]. Gradually advancing to target is recommended. IC should not be followed during this phase. However, over 3–4 days, this effect dissipates in most patients. Then, IC can be used to set targets. Another reason not to follow IC REE is administering non-nutritional calories (propofol, glucose, and citrate). Although not intended as nutrition, these calories contribute to energy intake and are substantial in individual patients [1]. When after commencement of nutrition, plasma phosphate drops below 0.65 mmol/l, known as refeeding hypophosphatemia (RFH), caloric restriction (< 500 kcal/day) is warranted. Studies have demonstrated increased mortality with high-calorie intake during RFH [1]. Therefore, then REE should not be the target.

## You will encounter practical issues—yes, probably

Large tidal volume variations during spontaneous mechanical ventilation can affect reliable measurements. Devices with a mixing chamber have better performance [10]. Early COVID-19 guidelines recommended against IC to reduce infection transmission as ventilator circuit disconnections may enhance aerosol production [11]. However, later studies using strict hygiene protocols show that safe application is feasible and provide crucial insights into metabolic changes of (hyper)metabolism over time [12].

Measuring REE during extracorporeal treatments (like CRRT and ECMO) poses significant challenges. Techniques may disturb REE by impacting temperature and stress levels. Moreover, techniques can also affect metabolism (adding bicarbonate and/or citrate) or non-pulmonary gas exchange. The effects of these techniques on REE are probably limited but also variable. Performing IC among these patients remains challenging [13]. Using complex equations to adjust for metabolic and gas exchange disturbances is possible but seems less feasible in daily practice [14].

## Indirect calorimetry is expensive—no, but there are costs involved

Costs of IC comprise device investment costs, consumables, calibration gas, and service. Additionally, staff time should be added. Typically, a procedure to measure takes 5–10 min. Modern interfaces are intuitive, and training during IC implementation can be limited to 1–2 h. Formal health economic evaluations are not available. Modern devices have acceptable costs per measurement (*personal communication: EdW, AvZ*). The business case is positive when improved outcomes reported in meta-analyses (mortality) translate into daily practice. Optimising nutritional performance may impact readmission rates, long-term functional outcomes (recovery from ICU-acquired weakness), and quality of life. Unfortunately, these endpoints have not been studied yet.

## Conclusions

Indirect calorimetry is the gold standard for measuring energy expenditure to set nutrition therapy goals during critical illness. Although recommended, the evidence underlying recommendations is limited. We have summarised when not to follow the REE from IC. Also, technical and patient-related limitations have been addressed. Future developments of IC to monitor REE continuously and during oxygen therapy and non-invasive ventilation are warranted to further enhance its application in daily practice.

## Data Availability

No datasets were used. Information on datasets and materials from studies reported can be found through the references.
